# Calcium and available phosphorus requirements for Japanese quails (*Coturnix japonica*) estimated by the factorial method

**DOI:** 10.1016/j.psj.2025.106059

**Published:** 2025-11-03

**Authors:** Danilo Vargas Gonçalves Vieira, Rannyelle Gomes Souza, Everton José do Nascimento Oliveira, Venúcia de Diniella dos Santos Bourdon, Thalita Rodrigues de Oliveira, Jossiel Santos Cruz, Adiel Vieira de Lima, Aline Beatriz Rodrigues, Fernando Guilherme Perazzo Costa, Matheus Ramalho Lima, Apolônio Gomes Ribeiro, Ricardo Romão Guerra, Lucas Rannier Ribeiro Antonino Carvalho

**Affiliations:** a Federal University of Northern Tocantins, Department of Animal Science, Araguaína, Tocantins, Brazil; b Federal University of Paraíba, Department of Animal Science, Areia, Paraíba, Brazil; c Federal Rural University of the Semi-Arid Region, Mossoró, Rio Grande do Norte, Brazil; d Department of Physiology and Pharmacology, Karolinska Institutet, Biomedicum 5B, Solnavägen 9, S-171 77, Stockholm, Sweden

**Keywords:** Available phosphorus, Calcium requirement, Comparative slaughter, Gain requirements, Prediction equation

## Abstract

Nutritional equations are essential tools for planning precise feeding strategies in poultry production. For Japanese quails (*Coturnix japonica*), early physiological maturity (around 40–45 days of age) and rapid mineral deposition during initial growth phases demand species-specific models. The factorial method enables accurate estimation of calcium and available phosphorus requirements by partitioning maintenance and growth needs, incorporating metabolic weight (kg⁰^.^⁶⁷) and nutrient utilization efficiency. These equations support sustainable and cost-effective feed formulation. To estimate calcium and available phosphorus requirements for Japanese quails during the starter (1–14 days) and grower (15–35 days) phases using predictive equations based on body weight, daily weight gain, and mineral retention efficiency. Two experiments were conducted with 655 female quails subjected to graded feeding levels (25 % to 100 % ad libitum). Maintenance requirements were determined via comparative slaughter and regression analysis between intake and retention. Gain requirements were estimated through serial slaughter and carcass composition analysis. Nutrient utilization efficiency was incorporated into the equations to calculate dietary requirements. Maintenance requirements increased with age: calcium from 60.86 to 223.58 mg/kg⁰^.^⁶⁷/day and available phosphorus from 43.18 to 106.35 mg/kg⁰^.^⁶⁷/day. Gain requirements were 24.91 mg/g/day (*Ca*) and 14.12 mg/g/day (*Ap*) in the starter phase, and 15.67 mg/g/day (*Ca*) and 8.48 mg/g/day (*Ap*) in the grower phase. Older quails showed higher mineral retention efficiency (71 % Ca; 73 % *P*), reducing dietary demand compared to previous studies. Reliable equations were developed: *Ca* = 60.86 × + 24.91 × *WG* (starter); *Ca* = 223.58 × *W ⁰^.^⁶⁷* + 15.67 × *WG* (grower). *Ap* = 43.18 × *W ⁰^.^⁶⁷* + 14.12 × *WG* (starter); *Ap* = 106.35 × *W ⁰^.^⁶⁷* + 8.48 × *WG* (grower). Where: *Ca* = Calcium requirements; *Ap* = Available phosphorus requirement; *W* = Body weight (kg); *WG* = Weight gain (g/quail/day). These equations enable precise diet formulation, optimize mineral use, and reduce environmental impact and production costs. They provide a technical foundation for nutritional programs tailored to Japanese quails.

## Introduction

Quail production, originally developed for various purposes such as hunting, meat, ornamentation, and egg production is now a reality on a global scale (Ribeiro et al., 2024). Countries like Spain, France, China, and the United States stand out in meat production, whereas China, Japan, and Brazil are prominent when quail breeding is aimed at egg production.

Whether for laying or meat production, quails exhibit early maturity, which is related to both growth rate and animal size (Tholon and Queiroz, 2011). Smaller animals typically exhibit faster growth and reach maturity earlier. Growth precocity is associated with the time required to reach sexual maturity and serves as a key parameter in genetic improvement programs, directly influencing nutritional requirements. In this context, models describing growth curves (Drumond et al., 2013; Mota et al., 2015; Grieser et al., 2018) support the premise that each species, lineage, or category of animal has distinct nutritional demands.

Comparisons of Gompertz growth curves for laying quails (Mota et al., 2015), meat quails (Drumond et al., 2013), light and semi-heavy commercial layers (Neme et al., 2006), and broilers (Demuner, 2016) reveal varying maturity rates (0.0720; 0.0694; 0.0245; 0.0411, respectively). Among these, Japanese quails exhibit the highest maturity rate, highlighting their distinct growth characteristics compared to other commercially raised birds.

According to Santos et al. (2014), Japanese quails have lower absolute (10.18 g) and relative (3.50 %) oviduct weights, and lower absolute (6.36 g) and relative (2.16 %) ovary weights, compared to commercial layers (Jardim Filho et al., 2008), which show an absolute oviduct weight of 76.98 g (6.58 % relative) and ovary weight of 36.04 g (3.08 % relative). However, the relative egg weight in quails is higher, reaching up to 10 % of their body weight. On average, quail eggs weigh around 12 g (Costa et al., 2008; Jordão Filho et al., 2011a; Vieira et al., 2012; Lima et al., 2014), whereas commercial layer eggs average approximately 65 g (Cardoso et al., 2014a; 2014b; Bezerra et al., 2015; Pastore et al., 2015). These peculiarities highlight the distinct nutritional needs of quails.

Due to the early maturity of quails (Drumond et al., 2016; Grieser et al., 2018), some authors have reported that using metabolic weight based on body surface area (kg^0.67^), rather than the traditional metabolic weight (kg^0.75^), is more appropriate for these birds (Resende et al., 2006). The exponent kg^0.67^ accounts for surface area differences in animals of varying masses and aligns better with their nutritional requirements (Dodds, Rothman and Weitz, 2001). Animals with different body weights do not have proportionally equal requirements; instead, metabolic weight provides a more accurate basis. Nóbrega (2018) demonstrated that when kg^0.67^ was used, increases in dietary energy concentration led to proportional increases in energy intake, body and egg energy retention, total retention, and heat production.

There are two primary methods for determining the nutritional requirements of birds: the traditional dose-response method and the factorial method. The factorial method predicts requirements through mathematical models that relate nutrient needs to metabolic weight, heat production, and metabolism. This approach allows the partitioning of total requirements into maintenance and production (or growth) needs and can incorporate environmental variables such as ambient temperature.

Historically, quail feeding programs were modeled after those of broilers and commercial layers. However, quails have been shown to require higher levels of protein and energy for maintenance, growth, and production. Additional factors influencing nutritional demands include climate, housing systems, species or lineage, and sex.

Several studies have evaluated the nutritional requirements of quails using the factorial approach (Silva et al., 2004a; 2004b; Jordão Filho et al., 2011a; 2011b; Mariz et al., 2017; Vieira et al., 2020a; 2020b; Broudon et al., 2023), though the literature remains limited. Therefore, specific models must be developed that consider species, sex, housing system, environmental conditions, and the animals’ growth curve characteristics.

## Materials and methods

The project was submitted to the Ethics Committee on Animal Use in Experimentation at the Federal University of Tocantins, Brazil, registered under number 23.101.00179/2017-53 and approved.

The experiments were conducted in the Coturniculture Sector of the Center for Agricultural Sciences at the Federal University of Northern Tocantins (UFNT), in Araguaína – TO, Brazil.

The techniques and methodologies used in this research are described in the book Research Methods in Monogastric Nutrition (Sakomura and Rostagno, 2016).

A total of 900 Japanese quails (*Coturnix japonica*) were acquired from Breeding Farm Vicami® at one day of age to estimate the maintenance and gain requirements during the initial phase (1-14 days) and the growth phase (15-35 days). To determine the calcium and available phosphorus requirements of Japanese quails, two experiments were conducted: one to estimate maintenance requirements and another to determine gain requirements. All birds remained healthy throughout the study, and no mortality was observed in either experiment.

A total of 655 female Japanese quails were used. The experiment was divided into two parts. The first phase (01-14 days of age) involved 350 quails with an initial average weight of 6.71±0.5 g/quail, of which 240 were placed in cages to estimate maintenance requirements, 75 for gain requirements, and 35 were slaughtered at one day of life as part of the reference slaughter for the initial phase. The second phase (15-35 days of age) involved 305 female quails with an initial average weight of 46.26±0.5 g/quail, with 240, 40, and 25 corresponding, respectively, to the total number of quails used to estimate maintenance requirements, gain requirements, and the reference slaughter group.

The quails were housed in galvanized wire cages (0.65 × 0.65 × 0.3 m), 420 cm²/quail, equipped with trough-type drinkers and feeders. The cages had a heating system (lamp) to maintain a thermal comfort zone of 35°C for the quails during the first week of life, with a light intensity of approximately 20 lux measured at the bird level. The experimental barn was equipped with curtains that were managed according to the ventilation needs within the facility.

The treatments consisted of four feed levels (100 %, 75 %, 50 %, and 25 %), with 100 % offered ad libitum. The 75 %, 50 %, and 25 % levels were calculated daily based on the ad libitum treatment, and water was provided ad libitum. The feed was formulated using corn and soybean meal (Table 1), meeting all the nutritional requirements of the quails in the initial and growth phases, as recommended by the Brazilian Tables for Poultry and Swine Nutrition (Rostagno et al., 2017).

**Table 1:** Chemical Composition and Percentage of Experimental Diets.

Ingredients (g/kg)	01-14 days of age	15-35 days of age
Corn	578.05	598.11
Soybean meal	368.62	360.82
Degummed oil	12.78	7.63
Dicalcium phosphate	22.07	17.49
Calcitic limestone	10.99	9.23
Common salt	4.83	5.06
DL-Methionine 99 %	1.61	1.13
L-Lysine HCl 78.4 %	0.63	0.05
L-Threonine 98.5 %	0.02	0.09
Premix†	0.40	0.40
Total	1000.0	1000.0
Nutrients (g/kg as fed)	01-14 days of age	15-35 days of age
Crude protein	212.8	210.9
Metabolizable energy (Mj/kg)	2.9	2.9
Calcium	10.92	9.11
Available phosphorus	5.13	4.28
Sodium	2.05	2.14
Potassium	8.60	8.52
Chlorine	3.71	3.74
Electrolyte balance (mEq/kg)	2045.0	2903.5
Digestible lysine	10.95	10.34
Digestible methionine + cysteine	7.44	6.93
Digestible threonine	7.33	7.34
Digestible valine	8.98	8.89
Digestible isoleucine	8.35	8.26
Digestible tryptophan	2.45	2.42
Digestible arginine	13.43	13.26
Digestible histidine	5.22	5.18
Digestible glycine + serine	17.34	17.16
Digestible phenylalanine + tyrosine	17.22	17.05
Digestible leucine	16.80	16.74

† **Composition per ton:** Manganese 18.1750 mg, zinc 17.500 mg, iron 11.250 mg, copper 2000 mg, iodine 187.50 mg, selenium 75 mg, vitamin A 1,400,000 IU, vitamin D3 300,000 IU, vitamin E 2.500 mg, vitamin K3 300 mg, vitamin B1 380 mg, vitamin B2 1,000 mg, vitamin B5 520 mg, vitamin B12 2,000 mg, folic acid 162.50 mg, pantothenic acid 2600 mg, niacin 7,000 mg, choline 71.593 mg, antioxidant additive 25.000 mg, halquinol 7,500 mg, salinomycin 16.500 mg.

The quails were divided into four experimental treatments in a completely randomized design, with six replications of ten quails each, totaling 240 quails per experimental phase (1-14 and 15-35 days of age).

The comparative slaughter method was used to estimate maintenance requirements. Of the 60 quails assigned to comparative slaughter, 35 quails were initially slaughtered at one day of age for the 1–14-day phase, while the remaining 25 quails were raised separately and slaughtered at 16 days to compose the reference group for the 15–35-day phase.

Before slaughter, the quails were subjected to a 12-hour solid fasting period and weighed to determine their initial and final weight after fasting. Slaughtering was conducted using the cervical dislocation method, aiming to prevent blood and feather loss, thus allowing accurate nutrient assessment in the carcass.

Carcasses were labeled with rearing phase, treatment, slaughter date, number of animals, and whether they belonged to maintenance or gain experiments, and stored in a freezer. Later, the carcasses were grounded three consecutive times using a "cutter" meat grinder, weighed, and placed in a forced ventilation oven at 55°C for approximately 72 hours for pre-drying.

Afterward, samples were processed twice more in a “cutter” mill and then in a “Willey” mill, ensuring homogeneous samples for chemical analysis. Dry matter and ash analyses were performed at the Animal Nutrition and Soil Laboratories of the Center for Agricultural Sciences at the Federal University of Northern Tocantins (UFNT), in Araguaina – TO, Brazil, following the Silva and Queiroz (2006) methodologies. Calcium and phosphorus analyses were conducted at the Soil Laboratory of the Federal University of Paraíba - Areia Campus, using the atomic absorption spectrophotometry method, as described by Tedesco et al. (1995).

The amount of calcium and phosphorus retained by the animals was calculated by determining the difference between the calcium and phosphorus present in the empty body of the quails in the final slaughter group and the reference slaughter group.

Maintenance requirements (mg/kg^0.67^/quail/day) were established through linear regressions of calcium and phosphorus retained in the carcass as a function of consumed calcium and phosphorus, using the SAS statistical package (version 9.0, SAS Institute Inc., Cary, NC, USA). The intercept of the regression line with the x-axis was interpreted as the dietary maintenance requirement, corrected for metabolic weight (MW). MW = [(P I_BW_ + P_FBW_) ÷ 2]^0.67^. P_IBW_ - Initial body weight, P_FBW_ - Final body weight and MW - metabolic weight. The regression coefficient (parameter “b”) indicates the efficiency of nutrient utilization.

The quails designated to establish calcium and phosphorus requirements for weight gain were raised on a floor covered with wood shavings. The area was equipped with 70 W incandescent lamps, drinkers, and feeders. Water and feed were provided *ad libitum* in this weight gain experiment. A digital thermo-hygrometer was used to measure temperature and humidity during the experimental period. The experimental diets were corn- and soybean meal-based (Table 1).

The calcium and phosphorus requirement for gain was determined through serial slaughtering to assess nutrient retention over the entire experimental period. 75 quails were used to estimate energy requirements for weight gain during the initial phase (1-14 days), with 15 quails slaughtered every three days (at 01, 03, 06, 09, 12, and 15 days of age). 40 quails were used in the growth phase (15-35 days), with 10 quails slaughtered every five days (at 15, 20, 25, 30, and 35 days of age).

Wheight gain requirements (mg/g/quail/day) were established through linear regressions of calcium and phosphorus retained in the carcass as a function of empty carcass weight. The regression coefficient (parameter “b”) indicates the net requirement for weight gain. The dietary requirement (DC) for daily weight gain was determined by relating the net requirement for weight gain to the utilization efficiency established in the maintenance experiment.

The errors were submitted to the Kolmogorov–Smirnov’s normality test (α = 0.01). The homogeneity of variances was evaluated by the Levene’s test (α = 0.01), and all variables showed a normal distribution of errors and homoscedasticity. Linear equations (α = 0.01) were estimated (SAS 9.0 - Proc Reg, SAS Institute Inc., 2004). All proposed models had a significant effect (t-test, α = 0.01) on the parameters of the equations ‘β_0_’; and ‘β_1_’.

Based on daily calcium requirements for maintenance and weight gain, a prediction model was developed to estimate calcium requirements for Japanese quails during the initial and growth phases, defined as: Ca (mg/quail/day) = Ca_m_ × P^0.67^ + Ca_g_ × WG. Where: Ca = Calcium requirement; Ca_m_ = Calcium requirement for maintenance (mg/kg^0.67^/day); Ca_g_ = Calcium requirement for gain (mg/g); *P* = Live weight (kg); WG = Weight gain (g/quail/day). Ap (mg/quail/day) = Ap_m_ × P^0.67^ + Ap_g_ × WG. Where: Ap = Available phosphorus requirement; Ap_m_ = Available phosphorus requirement for maintenance (mg/kg^0.67^/day); Ap_g_ = Available phosphorus requirement for gain (mg/g); *P* = Live weight (kg); WG = Weight gain (g/quail/day).

## Results and discussion

The average, minimum, and maximum temperatures and humidity observed during the experiment were 24.05± 0.07°C, 22.1 ± 0.07°C, and 35.20.08°C, respectively, with humidity values of 84.9 ± 0.31 %, 73± 0.34 %, and 95± 0.27 %.

It is observed that quails reduced their performance as the feed supply decreased (Table 2). This finding indicates that the methodology used to determine maintenance requirements was validated. The factorial methodology is based on the premise of considering the phases of the animal’s metabolism for the interpretation of maintenance requirements (Rostagno and Sakomura, 2016).

**Table 2:** Mean values of initial live weight (ILW, g/quail) and final live weight (FLW, g/quail), feed intake (FI, g/quail/day), weight gain (WG, g/quail/day), and ingested calcium (Ca_Ing_, mg/quail/day) and available phosphorus (Ap_Ing_, mg/quail/day) of Japanese quails according to Feed Supply Levels (FSL).

Maintenance Experiment (01 to 14 days of age)						
Age (days)	ILW	FLW	FI	WG	Ca_Ing_	Ap_Ing_
1st day						
Reference Slaughter	6.71	-	-	-	-	-
FSL (g/kg)	**ILW**	**FLW**	**FI**	**WG**	**Ca_Ing_**	**Ap_Ing_**
100†		46.26±0.59	8.73±0.25	2.64±0.02	95.35±2.78	44.79±1.30
75^‡^	-	34.97±1.16	6.47±0.06	1.88±0.01	70.69±0.60	33.21±0.28
50^‡^		24.33±1.33	4.39±0.03	1.17±0.03	47.96±0.32	22.53±0.15
25^‡^		12.89±0.77	2.28±0.11	0.41±0.02	24.92±1.23	11.71±0.58

Maintenance Experiment (15 to 35 days of age)						
Age (days)	ILW	FLW	FI	WG	Ca_Ing_	Ap_Ing_
15th day						
Reference Slaughter	48.40	-	-	-	-	-
FSL (g/kg)	**ILW**	**FLW**	**FI**	**WG**	**Ca_Ing_**	**Ap_Ing_**
100†		110.75±2.50	15.71±0.68	3.12±0.20	143.16±6.21	67.26±2.92
75^‡^	-	98.13±7.15	11.42±0.02	2.49±0.03	103.99±0.22	48.86±0.10
50^‡^		65.45±1.40	7.68±0.03	0.85±0.01	69.99±0.30	32.88±0.14
25^‡^		43.02±1.03	4.36±0.08	−0.27±0.04	39.72±0.70	18.65±0.33

† Animals that were fed ad libitum. ^‡^Animals that received a percentage relative to ad libitum consumption.

These data corroborate the findings of Silva et al. (2004), Jordão Filho et al. (2011), Nogueira et al. (2019), Vieira et al. (2020a), Vieira et al. (2020b), and Bourdon et al. (2023), who, when applying the factorial methodology to determine maintenance requirements, observed a decline in quail performance as feed intake decreased.

Nutritional requirements are influenced by intrinsic factors related to the animal, its environment, and interactions, as well as the feed itself; therefore, nutritional needs should not be considered as fixed values. The nutrient composition found in the animal depends on the diet’s quantity and nutritional content. Nutritional requirements, including protein, energy, and minerals, vary based on weight, growth rate, and production, but recommendations are typically based on intake levels as cited by Rostagno et al. (2017, 2024).

The animals subjected to the 25 % feed consumption treatment (Table 2) showed lower values of empty carcass weight, body calcium (Ca_c_) and phosphorus (P_c_), and retained calcium and phosphorus compared to those in the 100 % feed availability treatment. This outcome can be explained by calcium deficiency symptoms in growing birds, which include growth retardation, reduced feed intake, and bone fragility (Maiorka and Macari, 2002).

According to Li et al. (2017), for industrial poultry to fully express their genetic potential, they require the provision of optimal environmental conditions, which prioritize a well-founded nutritional regimen, specifically concerning mineral supply, including calcium and phosphorus.

The inclusion of calcium and phosphorus as dietary macrominerals in poultry farming is essential to drive various physiological processes. These include bone mineralization and integrity, acid-base balance, phospholipid and nucleotide formation, nervous function, and cellular energy metabolism (ATP), which are critical for sustaining growth, productivity, and overall bird well-being (Garcia et al., 2013; Zhang et al., 2020; Soheir et al., 2021; Omotoso et al., 2023; Sinclair-Black et al., 2023).

These statements corroborate the weight gain data (Table 2) of the quails in the treatment where they received only 25 % of the feed volume compared to the quails that fed at will. We observed a weight loss of 0.25 g/quail/day for quails in the growth phase (15 to 35 days of age).

The maintenance requirement of an animal is defined as the quantities of nutrients or energy necessary for its vital processes to remain normal, meaning, practically speaking, it applies when an animal does not undergo alterations in body composition (Rostagno e Sakomura, 2016).

Using the intake calcium and phosphorus (Table 2), calcium retention (Table 3), and phosphorus retention (Table 4) in the empty body of quails, regression equations were developed to estimate the daily calcium and phosphorus maintenance requirements (Table 5).

**Table 3:** Empty carcass weight (ECW, g/quail), average dry matter (DM, %), calcium content in the carcass (Ca, %), calcium converted to dry matter (Ca_DM_%), calcium content relative to body weight (Ca_C_, mg/g), retained calcium (Ca_ret_, mg/g/quail/day) of Japanese quails according to Feed Supply Levels (FSL).

Maintenance Experiment (01 to 14 days of age)						
Age (days)	ECW	DM	Ca	Ca_MS_	Ca_c_	Ca_ret_
1st day						
Reference Slaughter	6.71	24.18	2.72	0.66	44.18	-
FSL (g/kg)						
100†	46.26±0.59	25.51±0.57	3.69±0.29	0.94±0.60	435.39±25.03	27.94±1.79
75^‡^	34.97±1.16	25.02±0.97	3.44±0.09	0.86±0.03	301.25±17.51	18.36±1.27
50^‡^	24.33±1.33	24.75±0.27	4.02±0.20	0.99±0.06	241.90±20.04	14.12±1.43
25^‡^	12.89±0.77	20.82±0.32	4.60±0.27	0.96±0.05	123.41±11.71	5.66±0.83

Maintenance Experiment (15 to 35 days of age)						
Age (days)	ECW	DM	Ca	Ca_MS_	Ca_c_	Ca_ret_
15th day						
Reference Slaughter	48.4	26.7	2.72	0.73	351.05	-
FSL (g/kg)	ECW	DM	Ca	Ca_MS_	Ca_c_	Ca_ret_
100†	110.75±2.50	32.40±0.66	5.16±0.28	1.67±0.11	1852.65±126.1	75.08±6.31
75^‡^	98.13±7.14	28.82±1.99	4.47±0.20	1.29±0.11	1258.47±72.67	45.37±3.63
50^‡^	65.45±1.4	26.81±0.49	3.44±0.19	0.92±0.05	603.91±34.10	12.64±1.70
25^‡^	43.01±0.02	26.92±0.03	3.72±0.11	1.00±0.033	430.97±13.02	4.00±0.65

† Animals that were fed ad libitum. ^‡^Animals that received a percentage relative to ad libitum consumption.

**Table 4:** Empty carcass weight (ECW, g/quail), average dry matter (DM, %), phosphorus content in the carcass (P, %), phosphorus converted to dry matter (P_DM_%), phosphorus content relative to body weight (P_C_, mg/g), retained phosphorus (P_ret_, mg/g/quail/day) of Japanese quails according to Feed Supply Levels (FSL).

Maintenance Experiment (01 to 14 days of age)						
Age (days)	ECW	DM	P	P_MS_	P_c_	P_ret_
1st day						
Reference Slaughter	6.71	24.18	2.72	0.66	44.18	-
FSL (g/kg)	ECW	DM	P	P_MS_	P_c_	P_ret_
100†	46.26±0.59	25.51±0.57	1.93±0.07	0.49±0.015	227.69±7.26	14.41±0.52
75^‡^	34.97±1.16	25.02±0.97	1.97±0.03	0.49±0.016	171.97±6.45	10.43±0.46
50^‡^	24.33±1.33	24.75±0.27	2.08±0.09	0.52±0.03	125.51±9.68	7.11±0.69
25^‡^	12.89±0.77	20.82±0.32	2.26±0.07	0.47±0.01	60.64±4.43	2.48±0.32

Maintenance Experiment (15 to 35 days of age)						
Age (days)	ECW	DM	P	P_MS_	P_c_	P_ret_
15th day						
Reference Slaughter	48.4	26.7	2.72	0.73	351.05	-
FSL (g/kg)	ECW	DM	P	P_MS_	P_c_	P_ret_
100†	110.75±2.50	32.40±0.66	2.57±0.21	0.83±0.08	920.99±83.01	35.22±4.14
75^‡^	98.13±7.14	28.82±1.99	2.44±0.17	0.70±0.07	687.69±37.70	23.55±1.90
50^‡^	65.45±1.40	26.81±0.49	1.95±0.09	0.52±0.02	341.74±17.03	6.25±0.85
25^‡^	43.01±0.02	26.92±0.03	2.05±0.07	0.55±0.02	237.35±8.92	1.04±0.44

† Animals that were fed ad libitum. ^‡^Animals that received a percentage relative to ad libitum consumption.

**Table 5:** Regression equation for retained calcium (Ca_ret_, mg/P^0.67^) and phosphors (P_ret_, mg/P^0.67^/quail/day) as a function of ingested calcium (Ca_Ing_, mg/quail/day) and available phosphorus (Ap_Ing_, mg/quail/day), maintenance requirements calcium (Ca_m_, mg/P^0.67^/quail/day) and available phosphors (Ap_m_, mg/P^0.67^/quail/day), and utilization efficiencies for calcium and phosphorus (*k*, %) in Japanese quails from 01 to 14 days and from 15 to 35 days of age.

Equation	r^2^	MW†	Ca_m_	*k*
01-14 days				
Ca_ret_ = [(−1.62 ± 0.86) + (0.30 ± 0.01) × Ca_Ing_]	0.96	0.0875	60.86	30
15-35 days				
Ca_ret_ = [(−29.28 ± 3.8) + (0.71 ± 0.04) × Ca_Ing_]	0.94	0.1840	223.58	71
Equation	r^2^	MW†	Ap_m_	*kg*
01-14 days				
P_ret_ = [(−1.34 ± 0.32) + (0.35 ± 0.01) × Ap_Ing_]	0.98	0.0875	43.18	35
15-35 days				
P_ret_ = [(−14.31 ± 1.93) + (0.73 ± 0.04) × Ap_Ing_]	0.94	0.1840	106.35	73

† MW = [(P_IBW_ + P_FBW_) *÷* 2]^0.67^. P_IBW_ – Initial body weight, P_FBW_ – Final body weight; MW - metabolic weight.

Based on the average body weight of quails in the ad libitum feeding treatment, their metabolic weight (kg ^0.67^) was determined as 0.0876 for the 1–14-day phase and 0.184 for the 15–35-day phase.

The estimated maintenance calcium requirement for Japanese quails in the initial phase (01-14 days of age) was calculated using the equation: Ca_ret_ = [(−1.6183 ± 0.86) +(0.30371 ± 0.0131) × Ca_Ing_], adjusted r² = 0.96. Relative to the metabolic weight (0.0875), the maintenance calcium requirement was 60.86 mg/kg^0.67^/quail/day. Where: Ca_ret_ = retained calcium; Ca_Ing_ = ingested calcium from feed.

The estimated maintenance calcium requirement for Japanese quails in the growth phase (15-35 days of age) was calculated using the equation: Ca_ret_ = [(−29.2841 ± 3.77) + (0.71181 ± 0.039) × Ca_ing]_, adjusted r² = 0.94. Relative to the metabolic weight (0.184), the maintenance calcium requirement was 223.58 mg/kg^0.67^/quail/day. Where: Caret is retained calcium; Caing is calcium intake from feed.

Mariz et al. (2017) estimated calcium requirement, on average for the three temperatures studied (18, 24, and 28°C), the maintenance calcium requirement for quails aged 16-36 days as 176.24 mg/kg^0.75^/day at 24°C. The values reported in the present study differ from those previously found, estimating 223.58 mg/kg^0.67^/day for the growth phase. In Mariz’s (2017) study, the researcher employed a body mass-to-surface ratio of 3/4 (kg^0.75^), whereas the present study utilized a 2/3 (kg^0.67^) ratio.

If we used the interpretation of metabolic weight as kg^0.75^, we would estimate the calcium requirement for Japanese quails at 240.48 mg/kg^0.75^/day, 16.9 mg more calcium compared to 223.58 mg/kg^0.67^/day. In other words, we would recommend 7.6 % more calcium, which would imply a higher demand for limestone in the formulation and result in greater excretion of calcium and other minerals such as iron, magnesium, and zinc, which compete for the same intestinal absorption site (Straub, 2007). Absorption occurs in transcellular and paracellular pathways (Barboza et al., 2015).

Although calcium uses specific transporters, such as TRPV6 and PMCA1b (Peng et al., 2017; Liao et al., 2019; Ribeiro et al., 2024), which are different from those used by iron and zinc, its presence in high concentrations can interfere with the absorption of these minerals. This interference occurs mainly due to competition at intestinal transport sites, changes in solubility, and possible interactions with transporters such as DMT1 (Andrews, 1999), which is common to both iron and zinc. Therefore, even with distinct mechanisms, calcium negatively influences the bioavailability of iron and zinc in the gastrointestinal tract.

In Mariz’s (2017) experiments, the average temperature across the three rooms was 23°C, whereas in the present research, the average was 24°C. The higher requirement found (223.58 mg/kg^0.67^/day) is likely associated with improvements in the genetic quality of quails over time.

The estimated maintenance available phosphorus requirement (Ap_m_) for Japanese quails in the initial phase (01-14 days of age) was calculated using the equation: P_ret_ = [(−1.3395 ± 0.32) + (0.3545 ± 0.01) × Ap_Ing_], r² adjusted = 0.9801. Relative to the metabolic weight (0.0875), the maintenance available phosphorus requirement was 43.18 mg/kg^0.67^/quail/day. Where: P_ret_ = retained phosphorus; Ap_Ing_ = ingested available phosphorus from feed.

The estimated maintenance available phosphorus requirement (Ap_m_) for Japanese quails in the growth phase (15-35 days of age) was calculated using the equation: P_ret_ =[(−14.305 ± 1.92) + (0.731 ± 0.04) × Ap_Ing_], r² adjusted = 0.9368. Relative to the metabolic weight (0.184), the maintenance available phosphorus requirement was 106.35 mg/kg^0.67^/quail/day. Where: P_ret_ = retained phosphorus; Ap_Ing_ = ingested available phosphorus from feed.

Mariz et al. (2017) estimated available phosphorus requirement, on average, for the three temperatures studied (18, 24, and 28°C), the maintenance calcium requirement for quails aged 16-36 days as 178.26 mg/kg^0.75^/day at 24°C. The values reported in the present study differ from those previously found, estimating 106.35 mg/kg^0.67^/day for the growth phase. In Mariz’s (2017) study, the researcher employed a body mass-to-surface ratio of 3/4 (kg^0.75^), whereas the present study utilized a 2/3 (kg^0.67^) ratio, which may explain the differences in values observed across both investigations.

If we used the interpretation of metabolic weight as kg^0.75^, we would estimate the phosphorus requirement for Japanese quails at 130.46 mg/kg^0.75^/day, 24.11 mg more phosphorus compared to 106.35 mg/kg^0.67^/day. In other words, we would recommend 22.7 % more phosphorus, which would imply a higher demand for dicalcium phosphate in the formulation and result in greater excretion of phosphorus and other minerals such as iron, magnesium, and zinc, which compete for the same intestinal absorption site (Barlet et al., 1995).

Competition mainly occurs due to common transporters, such as NaPi-II cotransporters (phosphorus transporters), and other energy-dependent absorption mechanisms (Barlet et al., 1995). Additionally, the formation of insoluble complexes with these minerals can reduce their efficient absorption.

The use of kg^0.75^ in determining phosphorus recommendations for quail would have a more significant environmental impact compared to calcium. If kg^0.75^ were adopted, phosphorus requirements would increase by 22.7 %, whereas calcium requirements would grow by only 7.6 %. Additionally, this choice would raise the demand for dicalcium phosphate, increasing the cost of diet formulation, as phosphorus is the most expensive mineral to supplement. Another concerning factor is the high concentration of phosphorus in bird excreta, which poses a considerable environmental risk.

It is observed that the maintenance calcium requirement is significantly higher during the growth phase compared to the initial phase (223.58 vs. 60.86 mg/kg^0.67^/day, respectively), an increase of 267 %. Similarly, the maintenance available phosphorus requirement is significantly higher during the growth phase compared to the initial phase (106.35 vs. 43.18 mg/kg^0.67^/day, respectively), an increase of 146 %.

Considering that quails in the growth phase are undergoing skeletal development and can accumulate up to 80 % of the total calcium of an adult bird by the end of the first month of life (Edwards, 2000; Zeyad et al., 2020; Mohamed et al., 2024), we observed that the efficiency of utilizing dietary calcium and phosphorus is higher in older animals. This fact is linked to two factors: the gastrointestinal tract is more developed, and the bone tissue is in greater development. The more developed gastrointestinal tract improves digestion and nutrient absorption, while growing bone tissue demands more calcium and phosphorus.

Mariz et al. (2017) estimated the calcium maintenance requirements for Japanese quails aged 16-36 days under environmental temperatures of 18°C, 24°C, and 28°C. The values were 232.73, 139.56, and 158.88 mg/kg^0.75^/quail/day, respectively.

In the present study, the average temperature was 24°C, and maintenance was 223.58 mg/kg^0.67^/day. This shows that temperature influences the efficiency with which quails utilize dietary calcium for maintenance. The fact that we found higher values at the same average temperature compared to Mariz et al. (2017) may be associated with the genetics of the quails, possibly reflecting an evolution in the performance of more recent strains, indicating higher requirements, especially for maintenance. Additionally, the regular functioning of the organism at temperatures within the thermal comfort zone favors calcium metabolism. As a result, the effects of parathyroid hormone, calcitonin, and 1,25-dihydroxycholecalciferol (1,25(OH)₂D₃) in the intestine enable greater calcium deposition (Maiorka and Macari, 2002; Garcia et al., 2013; Zhang et al., 2020).

**Fig. 1:**
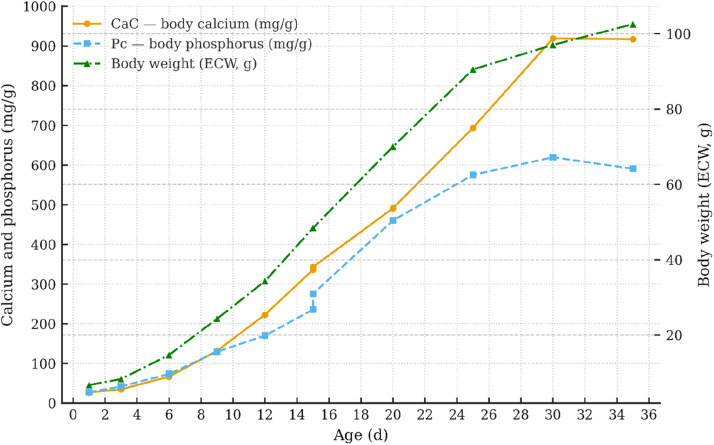
Relationship between empty carcass weight (ECW, g/quail) and mineral deposition (Cac – body calcium, mg/g; Pc – body phosphorus, mg/g) of Japanese quails at different ages.

Table 6 presents the average empty carcass weight (ECW) and the average quantities of calcium and phosphorus present in the carcass of Japanese quails, analyzed according to age-related weight gain. As age progresses, it is observed that the birds exhibited higher calcium and phosphorus concentrations within the carcass (Fig. 1), a phenomenon attributable to bone tissue development and the deposition of this mineral within the skeletal structure (Araújo et al., 2012; Soheir et al., 2021; Mahmoud et al., 2021).

**Table 6:** Empty carcass weight (ECW, g/quail), dry matter (DM, %), calcium and phosphorus content in the carcass (Ca, % - P, %), calcium e phosphorus converted to dry matter (Ca_DM,_ % - P_DM_, %), calcium and phosphorus content relative to body weight (Ca_C_, mg/g – P_C_, mg/g) in relation to the slaughter age of quails.

Growth experiment (01 to 14 days of age)								
Age (days)	ECW	DM	Ca	Ca_DM_	Ca_c_	P	P_DM_	P_c_
01	6.71	24.18	1.653	0.400	26.82	1.695	0.410	27.50
03	8.30	23.83	1.753	0.418	34.67	2.123	0.506	41.99
06	14.7	24.51	1.850	0.453	66.65	2.035	0.499	73.32
09	24.3	27.22	1.991	0.542	131.69	1.954	0.532	129.25
12	34.3	28.25	2.300	0.650	222.86	1.756	0.496	170.15
15	48.4	26.7	2.600	0.694	335.99	1.823	0.487	235.58

Growth experiment (15 to 35 days of age)								
Age (days)	ECW	DM	Ca	Ca_DM_	Ca_c_	P	P_DM_	P_c_
15	48.4	26.7	2.653	0.708	342.84	2.132	0.569	275.51
20	70.0	30.71	2.284	0.701	490.99	2.140	0.657	460.04
25	90.5	32.58	2.352	0.766	693.48	1.950	0.635	574.96
30	97.0	32.07	2.954	0.947	919.02	1.991	0.639	619.36
35	102.5	28.68	3.119	0.895	916.89	2.009	0.576	590.59

The estimated net calcium requirement (Table 7) for Japanese quails in the initial phase (01-14 days of age) was determined using the equation: Ca_c_ = (−33.843 + 7.4739 × ECW), adjusted r² = 0.992. The estimated requirement was 7.474 mg/g/quail/day. The dietary calcium requirement for the initial phase is 24.91 mg/g/quail/day, considering a 30 % calcium utilization efficiency in quails (Table 5). For the growth phase (15-35 days of age), the equation was: Ca_c_ = −236.16 + 11.126 × ECW, adjusted r² = 0.938. The estimated requirement was 11.126 mg/g/quail/day. The dietary calcium requirement for the growth phase was 15.67 mg/g/quail/day, considering a 71 % calcium utilization efficiency in quails (Table 5).

**Table 7:** Regression equation for body calcium (Ca_C_ – mg/g) and phosphorus (P_c_ – mg/g) as a function of empty carcass weight (ECW, g/quail), net calcium requirement for weight gain (Ca_g_ – mg/g/quail/day), net available phosphorus requirement for weight gain (Ap_g_ – mg/g/quail/day), calcium and phosphorus utilization efficiency (kg, %), and dietary calcium requirement for weight gain (DCa – mg/g/quail/day) and and dietary available phosphorus requirement for weight gain (Dap – mg/g/quail/day) in Japanese quails from 01 to 14 days and from 15 to 35 days of age.

Equation	r^2^	Ca_g_	*kg*	DCa
01-14 days				
Ca_c_ = - 33.843 + 7.4739 × ECW	0.99	7.474	0.30	24.91
15-35 days				
Ca_c_ = - 236.16 + 11.126 × ECW	0.94	11.126	0.71	15.67
Equation	r^2^	Ap_g_	*kg*	DAp
01-14 days				
P_c_ = 0.3579 + 4.9422 × ECW	0.99	4.942	35	14.12
15-35 days				
P_c_ = - 1.3273 + 6.1878 × ECW	0.95	6.188	73	8.48

The estimated net phosphorus requirement (Table 7) for Japanese quails in the initial phase (01-14 days of age) was determined using the equation: P_c_ = 0.3579 + 4.9422 × ECW, adjusted r² = 0.966. The estimated requirement was 4.942 mg/g/quail/day. The dietary phosphorus requirement for the initial phase is 14.12 mg/g/quail/day, considering a 35 % phosphorus utilization efficiency in quails (Table 5). For the growth phase (15-35 days of age), the equation was: P_c_ = −1.3273 + 6.1878 × ECW, adjusted r² = 0.953. The estimated requirement was 6.188 mg/g/quail/day. The dietary phosphorus requirement for the growth phase was 8.48 mg/g/quail/day, considering a 73 % phosphorus utilization efficiency in quails (Table 5).

Comparing the two ages in the present research, we found that younger quails have a higher calcium requirement. This may be related to the high demand for bone tissue formation or the low utilization efficiency during the initial phase (01-14 days of age). Therefore, it is essential to ensure a greater dietary intake to adequately meet nutritional recommendations.

Compared to other authors, Mariz et al. (2017) and Mariz et al. (2020) found weight gain calcium requirements of 28.15 mg/g/quail/day (Japanese quail) and 23.66 mg/g/quail/day (European quail), respectively. In the present study, we defined it as 15.67 mg/g/quail/day. In relation to the data from Mariz et al. (2017), there was a reduction of 44 %, and compared to Mariz et al. (2020), a reduction of 33.8 % when compared to the present study (15.67 mg/g/quail/day). The quails in the present study showed an efficiency of 71 % in absorbing calcium from the diet and depositing it into the carcass, while the data from the cited authors averaged 40 % efficiency. This explains the higher calcium dietary requirement found in the studies by the cited authors.

Compared to other authors, Mariz et al. (2017) and Mariz et al. (2020) found weight gain available phosphorus requirements of 9.70 mg/g/quail/day (Japanese quail) and 12.24 mg/g/quail/day (European quail), respectively. In the present study, we defined it as 8.48 mg/g/quail/day. In relation to the data from Mariz et al. (2017), there was a reduction of 12.58 %, and compared to Mariz et al. (2020), a reduction of 30.72 % when compared to the present study (8.48 mg/g/quail/day). The quails in the present study showed an efficiency of 73 % in absorbing phosphorus from the diet and depositing it into the carcass, while the data from the cited authors averaged 40 % efficiency. This explains the higher phosphorus dietary requirement found in the studies by the cited authors.

We observed that the efficiency of phosphorus utilization for both ages (01-14 and 15-35 days of age) of quails was higher, respectively 35 % and 73 % compared to 30 % and 71 % for calcium. This fact corroborates that, from a cellular and biochemical perspective, phosphorus is more intensively involved in daily metabolic reactions. While calcium also participates in important processes, it has a lower and more regulated metabolic demand, being stored primarily in bones rather than directly utilized (McDowell, 2003; Gutiérrez et al., 2020).

Such differences in calcium and phosphorus uptake efficiency could be attributed to variations in genetics, metabolism, or experimental conditions. The quails in the present study demonstrated superior efficiency in dietary calcium and phosphorus utilization, which directly impacts the lower dietary calcium and phosphorus requirements observed. These findings emphasize the need to consider species-specific and context-dependent factors when determining nutritional requirements, as well as the potential for optimizing diets to enhance resource efficiency.

Studies evaluating calcium requirements via dose-response methodology reveal substantial discrepancies in calcium and phosphorus levels both when comparing studies employing similar methodologies and when analyzing across different methodological approaches. There exists a significant gap in mineral nutritional requirement data for Japanese quails, and the limited data available exhibits inconsistencies, thereby necessitating further targeted research in this domain.

## Conclusion

The prediction equations developed to estimate calcium requirements for Japanese quails 01-14 days of age is: Ca (mg/quail/day) = 60.86 × W^0.67^ + 24.91 × WG. 15-35 days of age is: Ca (mg/quail/day) = 223.58 × W^0.67^ + 15.67 × WG. Where: Ca = Calcium requirement; *W* = Body weight (kg); WG = Weight gain (g/quail/day). The prediction equations developed to estimate available phosphorus requirements for Japanese quails 01-14 days of age is: Ap (mg/quail/day) = 43.18 × W^0.67^ + 14.12 × WG. 15-35 days of age is: Ap (mg/quail/day) = 106.35 × W^0.67^ + 8.48 × WG. Where: Ap = Available phosphorus requirement; *W* = Body weight (kg); WG = Weight gain (g/quail/day). These equations provide a practical tool for optimizing dietary formulations by allowing more precise adjustment of mineral levels according to the birds’ growth phase, thereby improving feed efficiency and reducing production costs.

## CRediT authorship contribution statement

**Danilo Vargas Gonçalves Vieira:** Writing – review & editing, Writing – original draft, Methodology, Investigation, Formal analysis, Data curation, Conceptualization. **Rannyelle Gomes Souza:** Investigation, Formal analysis, Data curation, Conceptualization. **Everton José do Nascimento Oliveira:** Formal analysis, Data curation, Conceptualization. **Venúcia de Diniella dos Santos Bourdon:** Formal analysis, Data curation, Conceptualization. **Thalita Rodrigues de Oliveira:** Formal analysis, Data curation, Conceptualization. **Jossiel Santos Cruz:** Formal analysis, Data curation, Conceptualization. **Adiel Vieira de Lima:** Writing – review & editing, Formal analysis, Data curation, Conceptualization. **Aline Beatriz Rodrigues:** Data curation, Conceptualization. **Fernando Guilherme Perazzo Costa:** Writing – review & editing, Writing – original draft, Visualization, Supervision, Project administration, Methodology, Formal analysis, Data curation, Conceptualization. **Matheus Ramalho Lima:** Writing – review & editing, Writing – original draft, Visualization, Methodology, Formal analysis, Data curation, Conceptualization. **Apolônio Gomes Ribeiro:** Writing – review & editing, Formal analysis, Data curation, Conceptualization. **Ricardo Romão Guerra:** Writing – review & editing, Formal analysis, Data curation, Conceptualization. **Lucas Rannier Ribeiro Antonino Carvalho:** Writing – review & editing, Formal analysis, Data curation, Conceptualization.

## Disclosures

The authors declare that they have no other conflicts of interest.

## References

[bib0001] Andrews N.C. (1999). Disorders of iron metabolism. N. Engl. J. Med..

[bib0002] Araújo G.M., Vieites F.M., Souza C.S. (2012). Importância do desenvolvimento ósseo na avicultura. Arch. Zootec..

[bib0010] Barboza G.D., Guizzardi S., Tolosa de Talamoni N. (2015). Molecular aspects of intestinal calcium absorption. World J. Gastroenterol..

[bib0003] Barlet J.P., Davicco M.J., Coxam V. (1995). Physiology of intestinal absorption of phosphorus in animals. Reprod. Nutr. Dev..

[bib0004] Bezerra R.M., Costa F.G.P., Givisiez P.E.N., Goulart C.C., Santos R.A., Lima M.R. (2015). Glutamic acid supplementation on low protein diets for laying hens. Acta Sci. Anim. Sci..

[bib0005] Bourdon V., Vieira D., Perazzo Costa F., Kaneko I., Lima A., Santana L., Neto E., Lima M., Rodrigues K., Vaz R., Cavalcante D. (2023). Modelo para predizer as exigências em proteína bruta para codornas japonesa em postura. Peer Rev..

[bib0006] Cardoso A.S., Costa F.G.P., Lima M.R., Nogueira E.T., Santos C.S., Sousa R.B., Lima R.C., Vieira D.V.G. (2014). Nutritional requirement of digestible threonine for white egg layers of 60 to 76 weeks of age. J. Appl. Poult. Res..

[bib0007] Cardoso A.S., Costa F.G.P., Silva J.H.V., Saraiva E.P., Nogueira E.T., Santos C.S., Lima M.R., Vieira D.V.G. (2014). Nutritional requirement of digestible tryptophan for white-egg layers of 60 to 76 weeks of age. J. Appl. Poult. Res..

[bib0008] Costa F.G.P., Rodrigues V.P., Goulart C.C., Neto R.C.L., Souza J.G., Silva J.H.V. (2008). Digestible lysine requirements for laying Japanese quails. Braz. J. Anim. Sci..

[bib0009] Demuner L.F. (2016). http://www.teses.usp.br/teses/disponiveis/74/74131/tde-03112016-140320/pt-br.php.

[bib0011] Dodds P.S., Rothman D.H., Weitz J.S. (2001). Re-examination of the “3/4-law” of metabolism. J. Theor. Biol..

[bib0012] Drumond E.S.C., Gonçalves F.M., Veloso R.C. (2013). Growth curve for quails. Rural Sci..

[bib0013] Garcia A.F.Q.M., Murakami A.E., Duarte C.R.A., Rojas I.C.O., Puzotti M.M. (2013). Use of vitamin D3 and its metabolites in broiler chicken feed on performance, bone parameters, and meat quality. Asian-Australas. J. Anim. Sci..

[bib0014] Grieser D.O., Marcato S.M., Furlan A.C., Zancanela V., Gasparino E., Del Vesco A.P., Lima N.C.F., Pozza P.C. (2018). Adjustment of nonlinear models and growth parameters and body nutrient deposition in meat-type and laying quail. R. Bras. Zootec..

[bib0015] Gutiérrez O.M., Porter A.K., Viggeswarapu M., Roberts J.L., Beck Jr G.R. (2020). Effects of phosphorus and calcium to phosphorus consumption ratio on mineral metabolism and cardiometabolic health. J. Nutr. Biochem..

[bib0016] Jardim Filho R.M., Stringhini J.H., Andrade M.A., Nunes A.B., Leandro N.S.M., Barcellos B. (2008). Egg quality, blood biochemical parameters and reproductive tract development for Lohmann LSL hens fed increasing levels of digestible lysine. Acta Sci. Anim. Sci..

[bib0017] Jordão Filho J., Silva J.H.V., Costa F.G.P., Sakomura N.K., Silva C.T., Chagas N.A. (2011). Prediction equations to estimate the demand of energy and crude protein for maintenance, gain and egg production for laying Japanese quails. Braz. J. Anim. Sci..

[bib0018] Jordão Filho J., Silva J.H.V., Silva C.T., Costa F.G.P., Sousa J.M.B., Givisiez P.E.N. (2011). Energy requirement for maintenance and gain for two genotypes of quails housed in different breeding rearing systems. Braz. J. Anim. Sci..

[bib0019] Li X., Zhang D., Bryden W.L. (2017). Calcium and phosphorus metabolism and nutrition of poultry: are current diets formulated in excess?. Anim. Prod. Sci..

[bib0020] Mahmoud A., Elwy A., Mohamed S., Ali M., Ezzat A. (2021). Effect of dietary calcium and phosphorus levels on growth, carcass characteristics, and liver and kidney functions of growing Egyptian geese. Poult. Sci..

[bib0021] Maiorka A., Macari M. (2002). Dietary vitamin or mineral mix removal during the finisher period on broiler chicken performance. J. Appl. Poult. Res..

[bib0022] Mariz C.B.L., Costa F.G.P., Vieira D.V.G., Lima M.R., Silva J.H.V., Jordão Filho J., Cavalcante D.T., Souza R.G., Bourdon V.D.S., Oliveira E.J.N., Cardoso A.S., Fernandes M.L., Ayres I.C.B., Nascimento D.S. (2020). Mathematical model for the prediction of available phosphorus and calcium requirements for European quails of 16–36 days old. Res. Soc. Dev..

[bib0023] Mariz L., Silva Filho J.J., Lima M.R., Costa P. (2017). P and Ca requirements for Japanese quail. J. Anim. Physiol. Anim. Nutr..

[bib0024] McDowell L.R. (2003).

[bib0025] Mohamed M.G., Metwally A.E., Mahmoud R.E., El-Gamal M.F. (2024). Impact of different levels of calcium and phosphorus in diet of broiler chickens on performance, carcass traits and blood parameters. J. Adv. Vet. Res..

[bib0026] Mota L.F.M., Alcântara D.C., Abreu L.R.A., Costa L.S., Pires A.V., Bonafé C.M., Silva M.A., Pinheiro S.R.F. (2015). Growth comparison of different genetic groups using nonlinear models. Braz. J. Vet. Res. Anim. Sci..

[bib0027] Neme R., Sakomura N.K., Fukayama E.H., Freitas E.R., Fialho F.B., Resende K.T., Fernandes J.B.K. (2006). Growth curves and deposition of body components in pullets of different strains. Braz. J. Anim. Sci..

[bib0028] Nóbrega I.P.T. (2018). https://repositorio.unesp.br/server/api/core/bitstreams/d76a9b9d-a8d0-4275-af52-3936acaeedf5/content.

[bib0029] Nogueira M.F.Z.F., Marcato S.M., Furlan A.C., Zancanela V., Finco E.M., Grieser D.O., Stanquevis C.E., Oliveira-Bruxel T.M. (2019). Models for predicting protein requirements for meat quail. Anim. Sci. J..

[bib0030] Omotoso A.O., Reyer H., Oster M., Maak S., Ponsuksili S., Wimmers K. (2023). Broiler physiological response to low phosphorus diets at different stages of production. Poult. Sci..

[bib0031] Pastore S.M., Gomes P.C., Barreto S.L.T., Viana G.S., Silva E.A., Almeida R.L., Barbosa L.V.B., Oliveira W.P. (2015). Nutritional requirement of digestible lysine for white-egg laying hens in production. Rural Sci..

[bib0032] Ribeiro A.G., Silva R.S., Silva D.A., Nascimento J.C.S., Souza L.F.A., Silva E.G., Ribeiro J.E.S., Campos D.B., Alves C.V.B.V., Saraiva E.P., Costa F.G.P., Guerra R.R. (2024). Heat stress in Japanese quails (Coturnix japonica): benefits of phytase supplementation. Animals.

[bib0033] Rostagno H.S., Albino L.F.T., Hannas M.I., Donzele J.L., Sakomura N.K., Perazzo F.G., Saraiva A., Teixeira M.L., Rodrigues P.B., de Oliveira R.F., de Toledo Barreto S.L., Brito C.O. (2017). https://edisciplinas.usp.br/pluginfile.php/4532766/mod_resource/content/1/Rostagno%20et%20al%202017.pdf.

[bib0034] Rostagno H.S., Albino L.F.T., Donzele J.L., Gomes P.C., Oliveira R.F., Lopes D.C., Ferreira A.S., Barreto S.L.T. (2024).

[bib0035] Sakomura N. K., H. S. Rostagno. 2016. Métodos de pesquisa em nutrição de monogástricos. 2nd edn. FUNEP, São Paulo, Brazil.

[bib0036] Santos I.C.L., Maciel W.C., Gomes V.S., Sampaio F.P., Machado D.N., Lima S.V.G., Lopes E.S., Silva R.C.R., Bezerra W.G.A., Teixeira R.S.C. (2014). Regression of the reproductive tract of European quails (Coturnix coturnix) submitted to forced molting by wheat bran diet. Acta Vet. Bras..

[bib0037] SAS Institute Inc (2004).

[bib0038] Silva D.J., Queiroz A.C. (2006).

[bib0039] Silva J.H.V., Silva M.B., Jordão Filho J., Rodrigues E.C., Costa F.G.P., Oliveira R.P. (2004). Protein and energy requirements for maintenance and gain in Japanese quails (Coturnix japonica) during 1 to 12 days of age. Rev. Bras. Zootec..

[bib0040] Sinclair-Black M., Garcia R.A., Ellestad L.E. (2023). Physiological regulation of calcium and phosphorus utilization in laying hens. Front. Physiol..

[bib0041] Soheir A., El-Shhat A., Awad A. (2021). Effect of dietary calcium and phosphorus levels on productive performance, carcass, and tibia characteristics of Sudani ducklings. J. Anim. Poult. Prod..

[bib0042] Straub D.A. (2007). Calcium supplementation in clinical practice: a review of forms, doses, and indications. Nutr. Clin. Pract..

[bib0043] Tedesco M.J., Gianello C., Bissani C.A., Bohnen H., Volkweiss S.J. (1995).

[bib0044] Tholon P., Queiroz S.A. (2011). Estimation of (co)variance components and genetic parameters for weights of red-winged tinamou using random regression models. Braz. J. Anim. Sci..

[bib0045] Vieira D.V.G., Oliveira E.J.N., Souza R.G., Bourdon V.D.S., Oliveira T.R., Silva K.E.C., Cruz J.S., Stivanin T.E., Souza T.A., Nascimento C., Rodrigues K.F., Vaz R.G.M.V., Lima M.R., Cavalcante D.T., Costa F.G.P. (2020). Mathematical models to predict Japanese quail crude protein requirements from 01 to 35 days old. Res. Soc. Dev..

[bib0046] Vieira D.V.G., Oliveira E.J.N., Souza R.G., Bourdon V.D.S., Oliveira T.R., Silva K.E.C., Cruz J.S., Stivanin T.E., Souza T.A., Nascimento C., Rodrigues K.F., Vaz R.G.M.V., Lima M.R., Cavalcante D.T., Costa F.G.P. (2020). Mathematical models to predict the energy requirements of Japanese quails from 01 to 35 days age. Res. Soc. Dev..

[bib0047] Zeyad K., Ahmad H., Hassan N. (2020). Response of broilers to calcium and phosphorus restriction: effects on growth performance, carcass traits, tibia characteristics, and total tract retention of nutrients. Ital. J. Anim. Sci..

[bib0048] Zhang L., He T., Piao X. (2020). Effects of normal and low calcium and phosphorus levels and 25-hydroxycholecalciferol supplementation on performance, antioxidant, meat quality, and bone properties of broilers. Poult. Sci..

